# Towards a Holistic Model of Care for Moral Injury: An Australian and New Zealand Investigation into the Role of Police Chaplains in Supporting Police Members following exposure to Moral Transgression

**DOI:** 10.1007/s10943-023-01908-2

**Published:** 2023-09-11

**Authors:** Andrea J. Phelps, Kelsey Madden, R. Nicholas Carleton, Lucinda Johnson, Lindsay B. Carey, Jean-Michel Mercier, Andrew Mellor, Jeffrey Baills, David Forbes, Peter Devenish-Meares, Fardous Hosseiny, Lisa Dell

**Affiliations:** 1 Phoenix Australia - Centre for Posttraumatic Mental Health, Department of Psychiatry, University of Melbourne, Level 3, 161 Barry Street, Carlton, VIC 3053 Australia; 2 Department of Psychology, University of Regina, Regina, Canada; 3 School of Psychology and Public Health, La Trobe University, Melbourne, VIC Australia; 4 Atlas Institute for Veterans and Families, Ottawa, ON Canada; 5 National Police Chaplaincy Group, Brisbane, Queensland Australia

**Keywords:** Moral injury, Police, Chaplains, Holistic approach, Religion, Spirituality, Biopsychosocial-spiritual

## Abstract

Police members can be exposed to morally transgressive events with potential for lasting psychosocial and spiritual harm. Through interviews with police members and police chaplains across Australia and New Zealand, this qualitative study explores the current role that police chaplains play in supporting members exposed to morally transgressive events. The availability of chaplains across police services and the close alignment between the support they offer, and the support sought by police, indicates they have an important role. However, a holistic approach should also consider organizational factors, the role of leaders, and access to evidence-based treatment in collaboration with mental health practitioners.

## Introduction

Policing is a highly dynamic occupation which involves exposure to potentially psychologically traumatic events (Carleton et al., 2019), occupational stressors (Carleton, Afifi, Taillieu, Carleton et al., 2020a), and ethical or moral dilemmas (Lentz et al., 2021). Ethical and moral dilemmas are particularly pertinent for policing which is based on codes of conduct and ethical standards grounded in morality and with a focus on ethics of care (Lentz et al., 2021). Police members are often required to make quick decisions in high risk and unpredictable environments, sometimes resulting in actions that go against their own moral code to protect themselves or others (Angehrn et al., 2020). More broadly there is increasing recognition that police members can be exposed to a range of events that transgress their moral values (Blumberg, 2022) and can be associated with poor mental health outcomes (Chan & Andersen, 2020; Demou et al., 2020; Simmons-Beauchamp & Sharpe, 2022).

The construct of moral injury (MI) has gained increasing attention from researchers and clinicians since Litz’s seminal paper (Litz et al., 2009), but has primarily been explored within a military and Veteran context to date (Papazoglou et al., 2020; Phelps et al., 2022). MI is not a mental health disorder, but can be associated with disorders such as posttraumatic stress disorder (PTSD) and major depressive disorder (Phelps et al., 2022). MI refers to the lasting psychological, social, and spiritual harms which may result from exposure to betrayal by authority in high-stakes situations (Bikos, 2023; Shay, 2014) or transgression of deeply held moral beliefs of right or wrong (Litz et al., 2009). For police, this might include behaving (or witnessing others behave) in a way that goes against personal values; for example, witnessing family violence or child exploitation.

These events are referred to as *potentially morally injurious events* (PMIEs) and fall on the severe end of a continuum of morally transgressive events (Litz & Kerig, 2019). At the other end of the continuum lies relatively minor and common *moral challenges* of daily life, and between the two extremes lies a range of mid-severity *moral stressors*. This range of morally transgressive events vary in their potential for lasting and impairing harm. Events on the less severe end of the continuum are more likely to be associated with transient feelings of moral frustration, whereas PMIEs increase likelihood of experiencing an enduring MI (Litz & Kerig, 2019). The aetiology of MI is exposure to a PMIE (Hall et al., 2022), but exposed individuals will be differentially impacted, with some unaffected by the event and others experiencing differing degrees of distress and other symptoms (Jones et al., 2022). Further, morally transgressive events experienced in an organisational setting can cause moral distress that, even at low levels, can be harmful for the individual, their work performance, and their organisational commitment (Chan & Andersen, 2020; Phelps et al., 2022).

MI may be distinct from PTSD with respect to neurophysiology (Barnes et al., 2019) and symptom profiles (Bryan et al., 2018), but the co-occurrence of MI and PTSD is high (Koenig et al., 2020). The concurrence makes intuitive sense given the probability of moral transgressions being associated with potentially psychologically traumatic events and therein posttraumatic stress injuries (Carleton et al., 2019), including PTSD. PMIEs exposures have also been associated with increased risk of symptoms related to major depressive disorder (Nazarov et al., 2018), generalized anxiety disorder (Lee et al., 2022), and self-injurious thoughts and behaviours (Jamieson et al., 2023; Zerach & Levi-Belz, 2019). As such, addressing the causes and outcomes of MI and moral distress is important, particular among police members who are on the frontline and are at high-risk of exposure to PMIEs.

Research into moral injury in policing is in its infancy, and greater understanding is of utmost importance for ensuring that police member wellbeing is at the forefront and that operational readiness is maintained. A whole-of-organisation approach is recommended to address moral injury that occurs in an organisational setting (Phelps et al., 2022), but chaplains play a potentially important part for several reasons. First, within a policing context, chaplains fulfil the role of offering informal care to staff and other members of the public who are both religious or non-religious, through various methods such as respectful listening, pastoral counselling, or, when appropriate, prayer (Layson et al., 2023). Moreover, police chaplains have the opportunity to support staff at all times, including before during and after critical incidents (Tunks Leach et al., 2020). Research showing that harm to one’s religious beliefs and spirituality may lead to poorer mental health outcomes among those exposed to PMIEs (Hall et al., 2022; Evans et al., 2018) supports the potential importance of chaplain-led pastoral models of care to address the spiritual impacts of moral transgressions (Carey et al., 2016; Koenig & Al Zaben, 2021). Lastly, the ready availability of chaplains within police organisations across Australia and New Zealand supports the feasibility of chaplains playing a key role. However, empirical evidence on the nature of support provided by chaplains in routine practice, and the extent to which that support is considered helpful by police members, is currently lacking.

## Aim

The current study was designed to use qualitative methods to capture the perspectives of both police members and police chaplains to better understand the current and potential role of chaplains in supporting police members exposed to PMIEs.

## Method

### Measures

MI outcomes were assessed using the Moral Injury Outcome Scale (MIOS; Litz et al., 2022). The MIOS is a cross-nationally developed, validated scale which includes 14-items measured on a 5-point scale which ranges from 1 (strongly disagree) to 5 (strongly agree). The MIOS produces a total score and two subscale scores, with higher scores indicating more severe moral injury. Total scores are defined as mild (14–28), moderate (29–42), or severe (43–56). Subscales measure (1) shame-related (i.e., self-related outcomes); and (2) trust-related MI outcomes (i.e., relating to others). The MIOS has good reliability and validity for the total score (α = 0.88), as well as for the shame-related (α = 0.86) and trust-related (α = 0.81) subscales (Litz et al., 2022).

### Procedure

Qualitative interview questions see Appendix 1 were developed by the project team in consultation with an advisory group comprising chaplains, mental health experts in MI, and people with lived experience. Interview questions for police members who had sought support following exposure to moral transgressions explored: PMIE exposures, sources of support that were accessed, anticipated outcomes, actual outcomes, and intended sources of support in the future. For police members who had not sought support, questions explored PMIE exposures, reasons for not seeking support, and intended sources of support for future help-seeking. Police members who did not mention chaplains as a source of support were explicitly prompted regarding willingness to engage with a chaplain for support. Interview questions for chaplains explored the types of PMIE for which police have sought chaplain support, the methods or approaches chaplains used to offer support, factors underlying selected methods of support, and observed outcomes (see Appendix 1).

Qualitative interview data were collected between February and September 2022. Police members and police chaplains were recruited through participating police services, using targeted emails and flyers, as well as social media advertisements. Police chaplains were also recruited through the National Police Chaplaincy Group. All advertising materials contained a link to the online screening survey that included the measures (for police members only), assessed eligibility, and obtained participant personal and demographic information. Prior to accessing the survey questions, participants were informed that clicking through to the next page indicated they consented to participate and have their data published in a journal article. Eligible police members were current serving and indicated that they had experienced a PMIE event according to the description of PMIEs in the MIOS. All chaplains currently employed (paid or volunteer) by a police service were eligible.

In an effort to collect a representative sample across Australia and New Zealand, the current study was designed to solicit four to five police member participants and two to three police chaplain participants from each Australian state, Australian territory, and New Zealand. Participants were contacted via phone or email, depending on their indicated preference, and were invited to attend an interview. Invitations to complete an interview were sequentially made to participants who expressed interest in participating in the study, except where several expressions of interest were submitted over a short period of time and there was an oversupply of potential participants from a particular area. In such cases, participants were selected based on their screening survey indicating a PMIE exposure or other disparate event relative to previously interviewed participants. Other participants were informed via email that they had been waitlisted for an interview. Some waitlisted participants were invited to attend an interview when other participants could not be contacted within three attempts. Interviewing continued until saturation was reached, with no new salient information emerging from interviews.

All interviews were completed via Zoom or Microsoft Teams and were audio recorded. Interviews typically lasted 30 to 60 min. De-identified audio recordings were sent to a confidential, Australian-based, third-party transcription service who adhered to the Commonwealth Privacy Act.

The current study was approved by the University of Melbourne Human Research Ethics Committee (reference number 2021-22399-22447-2). Further approval was sought from police services across all states of Australia and New Zealand and were subsequently provided by Western Australia (WA) Police Force Research Governance; Northern Territory (NT) Police, Fire and Emergency Services; Queensland (QLD) Police Service Research Committee; New South Wales (NSW) Police Force; Victoria (VIC) Police Research Coordination Committee; Australian Federal Police (AFP); Tasmanian (TAS) Institute of Law Enforcement Studies; and New Zealand (NZ) Evidence Based Policing Centre.

### Participants

A total of 121 police members and thirteen police chaplains completed the online expression of interest survey. There were 13 chaplains and 47 police members invited to attend a semi-structured qualitative interview. A total of 35 police members (see Table 1) and 11 chaplains (see Table 2) completed an interview.

### Data Analysis

Transcripts underwent thematic analysis via NVivo (version 12) using Braun and Clarke’s (2006) recommended methods. Transcripts were analysed by one researcher and a second researcher completed a fidelity check on 10% of the transcripts. Following the fidelity check, 70% of codes and themes were consistent between transcripts and over 80% agreement was achieved through discussion. The researchers familiarised themselves with the data before generating initial codes using an inductive approach. When half of the transcripts had been coded and grouped into initial themes, the data was presented to the study team and advisory group for input into how codes had been grouped to produce each theme. The remainder of the transcripts were coded and grouped into themes and subthemes and the final dataset was discussed with the study team and advisory group to ensure specifics of each theme were accurate and theme labels were refined. The MIOS description of PMIEs was used to classify events as PMIEs or less severe moral transgressions. Throughout the thematic analysis process, codes and themes were repeatedly cross-examined and refined to suit the developing narrative of the dataset.

## Results

### Demographics, Service-Related Characteristics

Police members and police chaplains were predominantly male (68.6% of police members and 81.8% of chaplains). The mean age of police members was 47.1 years and 60.7 years for police chaplains. Over half of police members (57.1%) had completed more than 20 years’ service and served in metropolitan locations (62.9%). All reported exposure to PMIEs on the MIOS, and the majority reported moral injury in the moderate to severe range (88% scored 29 or above on the MIOS). Police members were equally likely to have (40.0%) or not have (45.7%) a religious affiliation. Chaplains had also commonly served over 20 years (27.3%), though some had been in service for 5 years or less (27.3%). Most worked in metropolitan locations (58.3%), and they were predominantly Christian (90.9%).

**Table 1:** Police member (*n* = 35) demographics

Age	*M* = 47.1 years
Gender	
Male	68.6%
Female	28.6%
Prefer not to say	2.8%
**State**	
WA	14.3%
NT	5.7%
NZ	8.6%
AFP	31.4%
QLD	14.3%
VIC	22.9%
TAS	2.8%
**Position**	
Sub-Officer	34.3%
Officer	14.3%
Constable	51.4%
**Religious affiliation**	
Yes	40.0%
No	45.7%
Prefer not to say	11.4%
Not sure	2.9%
**Length of service**	
6–10 years	20.0%
11–20 years	22.9%
More than 20 years	57.1%
**Location**	
Metropolitan	62.9%
Regional	34.3%
Remote	2.8%
**MIOS**	
Mild MI	11%
Moderate MI	31%
Severe MI	57%

**Table 2:** Police chaplain (*n* = 11) demographics

Age	*M* = 60.7 years
**Gender**	
Male	81.8%
Female	18.2%
**State**	
AFP	18.2%
QLD	27.3%
VIC	27.3%
NSW	27.3%
**Length of service**	
Less than 1 year	9.1%
1–5 years	27.3%
6–10 years	18.3%
11–20 years	18.2%
More than 20 years	27.3%
**Religious affiliation**	
Christian (inclusive of all denominations)	90.9%
Did not disclose	9.1%
**Location**	
Metropolitan	58.3%
Regional	41.7%

*Participant experiences with moral stressors, PMIEs and subsequent help-seeking*

Police members described moral transgressions that varied along a continuum of severity (Litz & Kerig) (see Table 3). The severity of around half of these transgressions (55%) was consistent with a PMIE. Of note, level of distress did not necessarily correlate with severity of the moral transgression, and for the purpose of this study, the full range of moral transgressions were thus considered relevant. Moral transgressions could be aggregated into three categories: (1) 60% reported witnessing moral transgressions on the part of others; (2), 46% reported betrayal; and (3) 9% reported having perpetrated moral transgressions themselves. Around half (51%) reported having had several exposures to diverse moral transgressions during their police service. The majority of police members (66%) had sought support following exposure to moral transgression.

Chaplains reported that the most common types of PMIEs for which members sought their support were: (1) workplace and role related issues (e.g., having to reconcile other’s mistakes, male-female pay gap, mandatory vaccinations), reported by 64%; (2) deaths, reported by 55%; (3) incidents involving children, reported by 45%; and (4) motor vehicle accidents, reported by 36%. Note that the events reported by chaplains also represent moral transgressions of varying severity, including but not limited to, PMIEs.

**Table 3:** Themes and quotations relating to moral transgressions experienced by police

Police members	
**Witness**	
“I felt more force [was used] than was necessary.”	
“I came across a rape occurring.”	
**Betrayal**	
“We’re forced or coerced into taking action.”	
“I’ve been treated poorly by my organisation when I’ve asked for help, it hasn’t been provided.”	
**Perpetrate**	
“It was my responsibility and my personal view that I should intervene… but I wasn’t able to.”	
“I did wrong, I lost my temper.”	
**Police chaplains**	
**Workplace and role related issues**	
“[They have] issues of trust from within the organisation.”	
“[They have been] mistreated by the organisation.”	
**Deaths**	
“They’ve repeatedly had to go to fatalities”	
“[They have] experienced some of the emotional fallout of attending suicides.”	
**Incidents involving children**	
“Where there’s victims that are younger, particularly if it involves children.”	
“Particularly murder of children.”	
**Motor vehicle accidents**	
“[They] go to motor vehicle accidents.”	
“[They attend] a scene where you see dismembered bodies and body parts.”	

### Domains, Themes and Subthemes

**Fig. 1:**
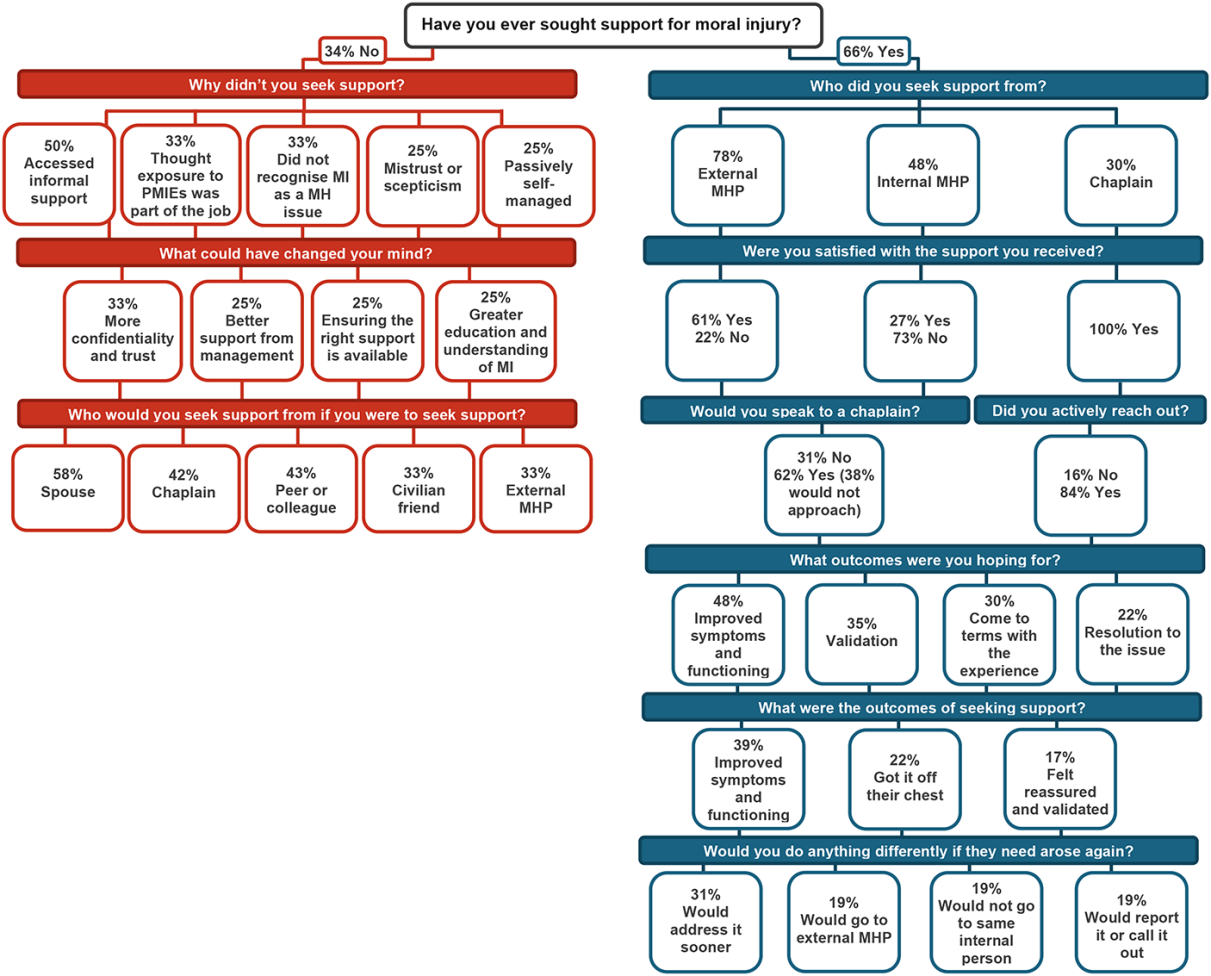
Flow chart of themes and subthemes for police members

The current study goals were addressed through five domains of enquiry for police members who had sought support, three domains of enquiry for police members who had not sought support (see Fig. 1), and six domains of enquiry for police chaplains. Themes and subthemes that emerged within each domain and are presented in Tables 4, 5 and 6 and summarised below.

**Table 4:** Domains, sub-domains, and themes for police members who had sought support

*Domain 4: Who did you seek support from?*	
*Sub-domain 4.1: Would you go to a chaplain?*	
**Theme: External mental health practitioner (78%)**	
“I started using EAP within a couple of weeks [of] that incident.”	
“I’ve had lots of external support.”	
**Subtheme: Felt they got the support they needed (61%)**	
“I did get everything I needed in the end.”	
“I would say yes… I’m self-aware of what I need and what I am looking for from care.”	
**Subtheme: Not supported as hoped (22%)**	
“I was essentially educated to believe that there were no answers.”	
“COVID shut that down. She closed [the psychology practice] up.”	
**Subtheme: Still getting support (17%)**	
“It’s continuing external to [the police service].”	
“There’s still some mess to work through.”	
**Theme: Internal mental health practitioner (48%)**	
“The psychologists that [the police service] had employed, they were very au fait with it.”	
“I have seen [mental health practitioners] internal.”	
**Subtheme: Not supported as hoped (73%)**	
“I didn’t – I received hardly any support.”	
“The psychs that are attached to the [police service], they have a very blanket view of things.”	
**Subtheme: Felt they got the support they needed (27%)**	
“Yeah, I think the department’s quite good with support.	
“Yes, from our health and welfare.” “”	
**Theme: Chaplain (30%)**	
“I went to the police chaplain.”	
“On multiple occasions [I got support] from a priest.”	
**Subtheme: Felt they got the support they needed (100%)**	
“[The chaplain] has supported me previously. I’m not religious, but she’s incredible.”	
“[The chaplain] just cared. Not because I was a police officer, just because I’m a human.”	
**Subtheme: Actively reached out to the chaplain (86%)**	
“[Needing to speak with a person] made me approach him.”	
“I usually reach out to the chaplain. I have known the chaplain for a very long time.”	
**Theme: Open to speaking to a chaplain (62% of participants assessed for this domain)**	
“I would probably go and talk to him because he seems really nice.”	
“Yes. I actually think that they have a really valuable space within the police force.”	
**Subtheme: Would only speak to a chaplain if they were approached by one (38%)**	
“If they were to approach me, I’d be open in liaising.”	
“I wouldn’t have initiated [a conversation].”	
**Theme: Would not speak to a chaplain (31% of participants assessed for this domain)**	
“I wouldn’t waste my energy trying to get a chaplain or a padre to support me.”	
“I feel awkward around them.”	
***Domain 5: When seeking support, what outcomes were you hoping for?***	
**Theme: symptoms to improve and increased functioning (48%)**	
“I knew I wasn’t coping and that I needed better strategies.”	
“To figure out what was going on with me because I had no idea what was going on.”	
**Theme: Validation (35%)**	
“Having the sounding board just to go, ‘am I crazy for thinking this yet again?’”	
“To reassure me that the reactions that I was having was within the normal sphere.”	
**Theme: To come to terms with the experience (30%)**	
“I just wanted to be me again.”	
“Recognition, understanding, support, some closure on [the experience].”	
**Theme: Resolution for the issue (22%)**	
“I had hoped that the issue would’ve been taken seriously, and it would’ve been resolved.”	
“I was hoping the system that’s causing moral injury would be looked at.”	
***Domain 6: What were the outcomes of seeking support?***	
**Theme: symptoms to improve and increased functioning (39%)**	
“I got a reduction in anxiety.”	
“My resilience and my tolerance have improved on a different level.”	
**Theme: Got it off their chest (22%)**	
“Being able to vent, to not bottle anything up, just get it all off the chest.”	
“A lot of it was just about listening. Me being able to unload.”	
**Theme: Felt reassured and validated (17%)**	
“Validation, reassurance, support.”	
“The outcome you get is the validation… you’ve acted in line with your morals and values.”	
***Domain 7: Would you do anything differently if they need arose again?***	
**Theme: Would do it differently (70%)**	
“I would now. Well, now I know what it is.”	
“I wouldn’t bother going down that avenue.”	
**Subtheme: Would address it sooner (31%)**	
“I probably should have taken time off earlier and had an extended break.”	
“have a lot more understanding about it now… I would see [the MI] earlier.”	
**Subtheme: Would go to an external mental health practitioner (19%)**	
“I would go straight to private psychologists and psychiatrists.”	
“I’d seek someone like [private psychologist], so someone independent of the organisation.”	
**Subtheme: Would not go to the same internal person again (19%)**	
“I wouldn’t go to [police service]. I wouldn’t go to any of my managers.”	
“I would not go through police welfare, I wouldn’t speak to a manager.”	
**Subtheme: Would report it or call it out (19%)**	
“I’d probably escalate it higher than what I did.”	
“Be upfront and just say, ‘listen, this is not acceptable,’ and be the noisemaker.”	

**Table 5:** Domains, sub-domains, and themes for police members who had not sought support

*Domain 1: Why didn’t you seek support*	
**Theme: Accessed informal support (50%)**	
“My wife and I support each other.”	
“I probably tend to rely more on friends and family.”	
**Theme: Did not recognise MI as a mental health issue (33%)**	
“I didn’t think that the way I was feeling was something that a psychologist could assist with.”	
“I didn’t specifically think that you could go and see a psychologist [for moral injury].”	
**Theme: Thinking exposure to PMIEs is part of the job (33%)**	
“[There was] expectation that it was part of the duties that people were told to do.”	
“You’re told… it’s just normal police work.”	
**Theme: Mistrust or scepticism (25%)**	
“There’s probably a degree of – large degree of – mistrust.”	
“I was sceptical as to whether another person could appreciate the quandaries that I was in.”	
**Theme: Passively self-managed (25%)**	
“[I] just deal with it internally.”	
“It’s just the way it is. Nothing will change. You just have to battle on.”	
***Domain 2: What could have changed your mind?***	
**Theme: More confidentiality and trust (33%)**	
“It is such a small area, [there’s] confidentiality issues.”	
“I mean confidentiality is important.”	
**Theme: Better support from management (25%)**	
“Being supported by the police hierarchy might add to that weight of feeling it’s okay.’	
“If that was to be supported… then it’s going to be a lot easier [to seek support].”	
**Theme: Ensuring the right support is available (25%)**	
“[Ensuring] they’ve selected the right people.”	
“I guess if the support was… more engaging.”	
**Theme: Greater education and understanding of MI (25%)**	
“Go read it on a board in a GP’s office and go, ‘hey, that’s me.’”	
“More discussion around moral injury as something that police experience.”	
***Domain 3: Who would you seek support from if you were to seek support?***	
***Sub-domain 3.1: Would you go to a chaplain?***	
**Theme: Spouse (58%)**	
“My wife would be my first port of call.”	
“Well family members definitely, because my husband is also in the job.”	
**Theme: Chaplain (42%)**	
“I’d take points from maybe a padre.’	
“I can speak to the chaplains.”	
**Theme: Peer or colleague (43%)**	
“Having colleagues that have felt similarly certainly does help.”	
“I would be most likely to seek support from a trusted peer.”	
**Theme: Civilian friend (33%)**	
“Perhaps a childhood friend.”	
“A friend of mine who’s an atheist. We share the same sort of values.”	
**Theme: External mental health practitioner (33%)**	
“[I] would probably use [the employee assistance program] as my initial go-to.”	
“I’d probably pick someone through the EAP, because again, it’s anonymous.”	

**Table 6:** Domains, sub-domains, and themes for police chaplains who have supported police members following exposure to moral transgression

*Domain 8: What approach do you take when helping a police member?*	
**Theme: Listen and provide a safe space (100%)**	
“I just try and listen to them.”	
“It’s all about listening for me. Letting them express their frustration, disappointment, hurt.”	
**Theme: Flexible spiritual guidance (91%)**	
“If they talk about a spiritual side, certainly we’ll engage with that.”	
“If they declare themselves from a Christian faith perspective, I’ll probably journey faster.”	
**Theme: Referral or encouragement to access support (73%)**	
“Part of our role is to help or assist members access [support], encourage them to do that.”	
“I’ll refer them to counselling if I think they need it.	
**Theme: Develop trust and rapport (73%)**	
“Once you’re known and trusted, they just go deep into telling you something.”	
“Any therapeutic relationship is based on trust and rapport.”	
**Theme: Encouragement and reinforcement (55%)**	
“What I’m hoping to achieve is empowering them to see… the difference they’re making.”	
“The mindset of ‘how can I direct this to a place of restoration, healing, or resolution?’”	
**Theme: Provide coping strategies (45%)**	
“Suggesting some fairly basic strategies for managing anxiety.”	
“I particularly value if they can have someone they can talk to, rely on, a friend… their partner.”	
**Theme: Offer a different perspective (36%)**	
“We can also help you maybe rethink what’s happened.”	
“Bring a little bit of different perspective and spread some light on the circumstances.”	
***Domain 9: What do you think this approach will achieve?***	
**Theme: Help them find a path to recovery (73%)**	
“Help them help themselves.”	
“It really is providing them with opportunity to find their pathway.”	
**Theme: Help members understand themselves (45%)**	
“Help them understand what is it out of their world view that’s been offended.”	
“It’s about helping people find the stuff that makes sense for them.”	
***Domain 10 What are the factors that determine the approach that you take?***	
**Theme: Spiritual or religious beliefs (64%)**	
“[Their religious beliefs] certainly does modify how you go about what you do.”	
“Unless [they] bring religion into the conversation, we don’t go there.”	
**Theme: Use a person-centred approach for everyone (55%)**	
“Most people say – ‘I’m not religious.’ I usually say, ‘that’s good, because I’m not either.’”	
“I want to deal with it on a personal and human level without bringing religious things into it.”	
***Domain 11: Are there certain factors that you would caution against?***	
**Theme: Too much of a religious approach (64%)**	
“Too much of a religious overlay.”	
“Coming on too strong from a religious mindset would be damaging.”	
**Theme: Thinking you’ve got all the answers (45%)**	
“I’m not assuming I can necessarily help them.”	
“I don’t go with any preconceived idea.”	
***Domain 12: What outcomes have you seen in police members seeking pastoral care?***	
**Theme: Relief from getting it off their chest (73%)**	
“That freedom to talk with somebody, that trust to be able to have that safe space.”	
“The feedback that I’ve got is… ‘it’s just good to talk to someone.’”	
**Theme: Move forward (36%)**	
“Helping them understand and help them deal with it and go forward.”	
“Some have been able to go back to work.”	
**Theme: They feel supported (36%)**	
“Feeling like someone’s genuinely interested in them.”	
“‘I couldn’t have done it without people like you just supporting, loving, encouraging.’”	

### Police Members who had Sought Support

The most frequently reported source of support (78%) was an external mental health practitioner (e.g., psychologists, counsellors, psychiatrists, Employee Assistance Programs). Mental health practitioners were not interviewed for the current study, making the nature of support provided unknown. Police members who had sought help from an external mental health practitioner variably felt they got the support they needed (61%), were not supported as hoped (22%), or were still getting support (17%). Approximately half (48%) of help-seeking police members went to an internal mental health practitioner. Of this group, most (73%) reported not being supported as they had hoped while the remainder (27%) felt they got the support they were hoping for. Approximately one third of help-seeking police members (30%) went to a chaplain, and all of this group reported feeling they got the needed support.

Help-seeking police members most commonly reported goals focused on improving symptoms and functioning (48%). Several help-seeking police members were seeking validation (35%) or hoping to come to terms with the experience (30%), with relatively fewer seeking resolution of the issue (22%). The police members reported outcomes of seeking support including improved symptoms and functioning (39%), feeling that they “got it off their chest” (22%), as well as feeling reassured and validated (17%).

Most police members (70%) reported their next help-seeking efforts would include addressing the stressor sooner (31%), going to an external mental health practitioner (19%), not going to the same internal person again (19%), and reporting the transgression (19%). Most (62%) were open to speaking to a chaplain but a third of that group would only do so if they were approached by the chaplain. In this context it is interesting to note that 86% of police members who had spoken to a chaplain initiated the contact themselves.

### Police Members who had not Sought Support

The most common reason (50%) police members gave for not seeking support was preference for using informal supports. Other reasons included not recognising MI as a mental health issue (33%), thinking that exposure to moral transgressions was just part of the job (33%), mistrust or scepticism (25%), or passively self-managing (25%). When asked what could have changed their mind about help-seeking, police members nominated the need for greater confidentiality and trust (33%), better support from management (25%), ensuring the right support is available (25%), and greater education and understanding of MI (25%).

Police members who had not previously sought support indicated future help-seeking would include speaking to a spouse (58%), a chaplain (42%), a peer or colleague (43%), a civilian friend (33%), or an external mental health practitioner (33%). Members who had not mentioned chaplaincy (*n* = 7) were specifically asked whether they would seek support from a chaplain in the future and almost all (*n* = 6) indicated that they would.

### Police Chaplains

Police chaplains reported using several support strategies for police exposed to moral transgressions. All reported listening and providing a safe space for police members and almost all (91%) reported using flexible spiritual guidance rather than no spiritual focus. Most chaplains (73%) endorsed referral or encouragement to access support, as well as developing trust and rapport (73%), as important components of their role. Approximately half (55%) of chaplains reported offering encouragement and reinforcement of actions being taken or providing coping strategies (45%), and several reported offering a different perspective (36%).

Police chaplains reported that their goal in supporting police members was to facilitate member recovery pathways (73%) or increased member self-awareness about why a given event may have impacted the member (45%). Approximately half of chaplains reported using a person-centred approach for everyone (55%), and more than half indicated each member’s spiritual or religious beliefs impacted their approach (64%). Chaplains felt that it was unhelpful to take too much of a religious approach (64%), or think you’ve got all the answers (45%). Chaplains reported outcomes including having seen police members experience relief from “getting it off their chest” (73%), moving forward (36%), and having a sense of feeling supported (36%).

## Discussion

Several previous studies have reported on MI interventions developed and delivered by chaplains (see the scoping review from Jones et al., 2022 and Currier et al., 2023) but we believe that this is the first to use a qualitative methodology to explore the support provided by chaplains in routine practice and how this support is received by police members. The initial scope of enquiry was the nature of support sought and provided following the experience of a PMIE but as noted in the results, the types of moral transgressions described by both police members and police chaplains varied on a continuum of severity, which included, but was not limited to, PMIEs. For the purpose of this study, the full continuum of moral transgressions was considered relevant, with the severity of impact, as measured by the MIOS, unrelated to the severity of the event. Incidentally, this is not dissimilar to studies in the PTSD field that have found PTSD severity to be unrelated to severity of the Criterion A stressor, or indeed to be more severe in some cases following a non-Criterion A stressor (Gold et al., 2005; Long et al., 2008). It is beyond the scope of this paper to explore this in depth, but the finding highlights the need for further research into the impacts of moral transgressions beyond those considered PMIEs.

The most common type of moral transgression reported by police members involved witnessing another person act in a way that transgressed the individual’s beliefs of right and wrong or feeling betrayed. Such moral transgressions were not uncommon with half of respondents reporting exposure to several such events. Support was most commonly sought from mental health practitioners outside of the police service. Satisfaction was markedly higher for external mental health practitioners (relative to internal mental health practitioners). The discrepant satisfaction reports are partially explained by police members feeling a greater sense of confidentiality and trust when accessing an external health service provider. Indeed, participants reported feeling like they could talk about things more freely and be open and honest when seeking support externally to their organisation. Through facilitating confidentiality, external health service providers may enable trust. This may also be true for chaplains who use approaches which aim to facilitate trust (Tunks Leach et al., 2023). This was supported by the current findings whereby police felt that chaplains were trusting, understanding, and made police feel cared for. This facilitation of trust has the potential to be particularly beneficial for police members who had experienced betrayal-based moral stressors and PMIEs and had lost trust in their organisation.

Only a third of police members sought support from chaplains. A preference for support from mental health practitioners over support from chaplains has been noted in previous research with public safety personnel (Carleton, Afifi, Turner et al., 2020). However, a notable finding in the current study was that satisfaction with support was highest for chaplains. All police who sought support from chaplains reported being satisfied with the support they received. This may be attributed to close alignment between support that police members indicated they were looking for (have feelings validated and come to reconcile their experience) and the support that chaplains indicated they provide (listening, providing safe spaces, and seeking to validate members’ feelings). It is also possible that police members with more severe symptoms were more likely to seek support from mental health practitioners than those who sought support from chaplains and that symptom severity had an influence on satisfaction with support. This was not tested in the current study.

The potential importance of chaplains in providing support was further highlighted by evidence that, among police members who had not yet sought support, most were open to speaking with a chaplain for future support, preferring the chaplain to initiate the approach. This result suggests greater chaplaincy outreach could be an effective proactive approach to mitigating MI and supporting mental health. There was also a small proportion of members who were not open to chaplaincy support, the reasons for which warrant further attention.

While the support currently provided by chaplains aligns closely with what police members say that they need, this should be considered one component of an holistic approach that also involves evidence-based care from mental health practitioners for MI and related mental health injuries such as PTSD (Koenig et al., 2020), major depressive disorder (Nazarov et al., 2018), generalized anxiety disorder (Lee et al., 2022), as well as self-injurious thoughts and behaviours (Zerach & Levi-Belz, 2019). It has been previously reported that police chaplains can potentiate beneficial spiritual interventions for directly improving MI outcomes (Currier et al., 2023) as well as facilitating access to psychological services as a function of regular, direct engagements with members and outreach opportunities. Both of these were observed in the current study with the majority of chaplains describing their approach as offering flexible spiritual guidance alongside referral or encouragement to access other forms of support, thus demonstrating an appreciation of their role within the holistic model of care.

Although not a key focus of the current research, it was noteworthy that only 2 in 5 police members reported improvement in their symptoms or level of functioning after seeking support from either an internal or external mental health practitioner or a chaplain. We have a significant challenge ahead to develop, evaluate and implement organisation-wide and evidence-based approaches to prevention and support for workers likely to be exposed to moral transgressions in the course of their work (Phelps et al., 2022).

### Strengths and Limitations

The current study has several strengths and limitations that contextualise the results and can help inform future research. The qualitative methodology was an important strength, providing more nuanced insights about the unique moral stressors and PMIEs experienced by police members and chaplains, as well as the help-seeking attitudes and behaviours of police members. With better understanding of police member and police chaplains’ experiences, attitudes and behaviours, future research using quantitative methodologies will strengthen and broaden this understanding and allow for comparison between different demographic groups within the police force. The diversity of study participants may have been limited by using a recruitment strategy which included promotion through chaplains. The recruitment strategy may have facilitated a sampling bias for police member participants who were open to chaplaincy, though promotion through other channels such as social media and advertisements hopefully helped to mitigate the likelihood of bias. Future research using broader recruitment strategies may help mitigate against such biases and produce more representative results. The participants were also predominantly older men, which does not accurately reflect contemporary police demographics that include a higher proportion of young women members. This warrants additional research with a larger cohort of both police members and police chaplains.

## Conclusion

Despite the limitations, the results provide evidence that police members experience frequent exposures to moral transgressions associated with mental health challenges, and highlight the importance of police organisations addressing MI. The current results suggest four potential foci. First, in addition to the traditional focus on mental health service providers, organisations should recognise the potential role of chaplains in supporting members who have experienced PMIEs and facilitating access to mental health service providers. Second, many police members did not recognise that exposure to moral transgressions as potentially injurious, highlighting the importance of increasing awareness and education about risks, symptoms, and sources of support. Third, several of the moral transgressions that were reported were associated with organisational factors, and participating police members suggested resolutions required organisational change. The feedback highlights the importance of recognising the impact of organisational culture, policies, and practices, on individual member wellbeing. Finally, participating police members reported that perceiving higher levels of confidentiality and trust, better supports from management, and access to the right supports, would all have facilitated their help-seeking. The suggestions underscore the importance of organisational strategies to promote help-seeking through supportive management, and ready access to evidenced-based, high quality, and confidential holistic care.

In sum, this research contributes to our increased appreciation that wellbeing strategies within policing jurisdictions need to accommodate a biopsychosocial-spiritual framework to assist police members in looking after their holistic wellbeing, as the impacts and challenges of morally injurious events are having a significant cost and impact on policing across Australia and New Zealand.

## Appendix 1

### Questions for chaplains

1. Does your understanding of moral injury line up with this or is it different?
2. Can you share what types of events that police members have spoken about with regard to moral injury?
  *Please don’t provide any details of names or dates, I am just interested in the types of events.*

- Prompt: do police members approach you about such events or do you approach police members?

3. What approach or approaches do you take when helping a police member who has experienced a moral injury?

- Reminder: ensure they share what they think that approach will achieve/influence.

4. What are the factors that determine the approach that you take?
  *E.g., religious, or spiritual beliefs.*

5. Are there certain approaches you have found not to be helpful and you would caution against?
6. What outcomes (positive or negative) have you seen in police members seeking pastoral care in the context of moral injury?

### Questions for police members

1. In one or two sentences, could you please tell me what has been your personal experience involving moral injury?
  *Please don’t provide any details of names or dates, I am just interested in the situation in general terms.*
2. Have you ever sought support for moral injury?

**If YES**:

3. When seeking support with moral injury, what outcomes were you hoping for?
4. Who did you seek support from and why?

- Prompt: if you received support from a Chaplain, did you actively seek that support or were you approached by a Chaplain?

6. What did that support involve?

- Prompt: how long were you engaged in support?

6. What was the outcome or outcomes?
7. Did you receive all the support you needed and for as long as you needed?

- Prompt: were you left with any unmet needs?

8. Would you do anything different if the need arose again in the future to seek support for moral injury?

- Prompt: would you seek support from a Chaplain or would you be open to speaking to a chaplain if they were to approach you? Why/why not?

**If NO**:

3. Why is that?
4. What, if anything, could have changed your mind so that you would have sought support?
5. If you were to seek support, who do you think you would be most likely to see and why/why not?
6. E.*g., mental health practitioner.*

- Prompt: did you speak to an internal or external mental health practitioner, chaplain, peer, family member etc.
